# Concurrent Mentions of Vaping and Alcohol on Twitter: Latent Dirichlet Analysis

**DOI:** 10.2196/51870

**Published:** 2024-11-12

**Authors:** Lynsie R Ranker, David Assefa Tofu, Manyuan Lu, Jiaxi Wu, Aruni Bhatnagar, Rose Marie Robertson, Derry Wijaya, Traci Hong, Jessica L Fetterman, Ziming Xuan

**Affiliations:** 1 Community Health Sciences Boston University School of Public Health Boston, MA United States; 2 Department of Computer Science Boston University Boston, MA United States; 3 Annenberg School of Communication University of Pennsylvania Philadelphia, PA United States; 4 Department of Medicine University of Louisville Louisville, KY United States; 5 American Heart Association Tobacco Regulation and Addiction Center Dallas, TX United States; 6 Department of Medicine Vanderbilt University Nashville, TN United States; 7 Monash University Jakarta Indonesia; 8 College of Communication Boston University Boston, MA United States; 9 Evans Department of Medicine and Whitaker Cardiovascular Institute Boston University Chobanian & Avedisian School of Medicine Boston, MA United States

**Keywords:** e-cigarettes, alcohol, social media, vape, tweet, vaping, alcohol use, co-use, substance use disorder, social networking site, insight, regulation, youth, vaping policy

## Abstract

**Background:**

Co-use of alcohol and e-cigarettes (often called vaping) has been linked with long-term health outcomes, including increased risk for substance use disorder. Co-use may have been exacerbated by the COVID-19 pandemic. Social networking sites may offer insights into current perspectives on polysubstance use.

**Objective:**

The aims of this study were to investigate concurrent mentions of vaping and alcohol on Twitter (subsequently rebranded X) during a time of changing vaping regulations in the United States and the emergence of the COVID-19 pandemic.

**Methods:**

Tweets including both vape- and alcohol-related terms posted between October 2019 and September 2020 were analyzed using latent Dirichlet allocation modeling. Distinct topics were identified and described.

**Results:**

Three topics were identified across 6437 tweets: (1) flavors and flavor ban (n=3334, 51.8% of tweets), (2) co-use discourse (n=1119, 17.4%), and (3) availability and access regulation (n=1984, 30.8%). Co-use discussions often portrayed co-use as positive and prosocial. Tweets focused on regulation often used alcohol regulations for comparison. Some focused on the perceived overregulation of vaping (compared to alcohol), while others supported limiting youth access but not at the expense of adult access (eg, stronger age verification over product bans). Across topics, vaping was typically portrayed as less harmful than alcohol use. The benefits of flavors for adult smoking cessation were also discussed. The distribution of topics across time varied across both pre– and post–regulatory change and pre– and post–COVID-19 pandemic declaration periods, suggesting shifts in topic focus salience across time.

**Conclusions:**

Co-use discussions on social media during this time of regulatory change and social upheaval typically portrayed both vaping and alcohol use in a positive light. It also included debates surrounding the differences in regulation of the 2 substances—particularly as it related to limiting youth access. Emergent themes from the analysis suggest that alcohol was perceived as more harmful but less regulated and more accessible to underage youth than vaping products. Frequent discussions and comparisons of the 2 substances as it relates to their regulation emphasize the still-evolving vaping policy landscape. Social media content analyses during times of change may help regulators and policy makers to better understand and respond to common concerns and potential misconceptions surrounding drug-related policies and accessibility.

## Introduction

Since 2014, nicotine-containing products such as e-cigarettes (often referred to broadly as vaping) have been the most used tobacco product among youth in the United States [1]. An estimated 14% of US high schoolers currently vape [2], while nearly one-third report consuming alcohol in the past 30 days [1]. Alcohol and vaping can often co-occur. A recent meta-analysis found that vaping was associated with a 6-fold increased odds of alcohol consumption as well as binge drinking or drunkenness [3]. Nicotine, the addictive chemical found in e-cigarettes, activates pathways in the brain that may reinforce addictive behavior and has been found to enhance the pleasurable effects of alcohol consumption as well as increase cravings [4,5]. A recent study using ecological momentary assessment found that youth who reported co-use of e-cigarettes and alcohol were more likely to report high-risk behaviors, including binge drinking, and that co-use was more common in social contexts [6].

Polysubstance use is concerning to clinicians and public health practitioners, as it may increase the risk of adverse long-term health and social outcomes including increased substance use disorder and reduced educational attainment [7-11]. Substance use and addiction concerns have been further exacerbated by the COVID-19 pandemic, which led to increased mental health strain and alcohol use [12]. While research on the long-term health effects of e-cigarettes is still emerging, addiction to alcohol and tobacco has been found to have health consequences including increased cancer risk and exacerbation of mental health disorders [13,14]. For young people, exposure to nicotine is linked to detrimental effects on learning, memory, and attention [15,16]. Further, addiction to nicotine and alcohol can stress relationships, increase feelings of isolation, and increase the risk for injuries and mortality [13,17].

Youth today report being on the web “near constantly” and spend the majority of that time on social networking sites such as YouTube, Twitter (subsequently rebranded X), and Instagram [18]. On these platforms, they are increasingly exposed to substance use–related content generated by peers, influencers, or businesses [19-24]. In one recent survey, nearly three-quarters of middle and high school youth who used social media reported seeing e-cigarette–related content on these platforms [25]. Social learning theory posits that individuals learn vicariously through observing and modeling the behaviors of others [26]. Thus, social media portrayals, which commonly place substances in a positive light, could create or reinforce positive use expectancies by directing attention to youth-appealing content, encouraging modeling of this behavior on their own accounts, and increasing motivation for such behaviors by portraying them as part of a social norm. Indeed, exposure to substance use content on social media is linked with positive use expectancies, norms, and initiation [27-29], and prior work has found associations between exposure to alcohol content on social media and binge drinking among college students [30,31].

One commonly used social networking platform is Twitter. It is a microblogging platform that, as of early 2022, had an estimated 229 million daily active users [32]. Individuals aged 18-49 years are most likely to engage in vaping, and they also represent an estimated 76% of Twitter’s active users [33]. The text-based, timely nature of tweets offers an opportunity to explore social media discourse, and analyses of posts using methods such as content analysis may provide valuable insight into individual’s perceptions and experiences.

Content analyses of tweets have been used to understand a variety of substance use–related topics, including e-cigarette perceptions [34], e-cigarette policy reactions [35], addiction concerns during COVID-19 [36], and e-cigarette cessation campaign responses [37]. In addition, such approaches have been applied to the analysis of other social media forums [38,39]. Findings from this body of literature have helped inform health communication and intervention efforts by assessing the current understanding and viewpoints of potential audiences [35,40]. Analyses have also been conducted to monitor brands that are increasingly using social media as a marketing tool—potentially in violation of rules surrounding marketing to underage youth who use these platforms frequently [38,41,42]. While content analyses have historically relied upon human coding, the application of machine learning techniques to identify emergent topics related to substance use is becoming more common in order to analyze large amount of social media data concurrently [43-45]. Yet, few studies have applied these tools to examine polysubstance use discussions on social media [46,47]. Furthermore, none have examined such social media conversations in times where mental health, lifestyles, and substance use behaviors may be in flux.

The emergence of the COVID-19 pandemic in the United States was a time period characterized by social upheaval as well as key federal tobacco regulatory changes. Specifically, the federal Tobacco 21 law prohibiting sales of tobacco products to those younger than 21 years of age was implemented on December 20, 2019 [48], and the federal ban prohibiting the sale of characterizing flavors (excluding tobacco and menthol) in cartridge-based e-cigarettes was implemented on January 2, 2020 [49]. While several states (eg, Hawaii, California, and Oregon) had already increased the purchase age from 18 to 21 years or placed restrictions on flavored products, both of these federal US-wide policies were major updates to the regulation of tobacco in the United States.

The goal of this study was to apply a computational content analysis method to characterize the common topics—or themes—of a sample of tweets that include both vaping-related and alcohol-related terms. We specifically focus on a time period of upheaval in the United States by using a dataset, which includes tweets during both (1) the emergence and initial peaks of COVID-19 in the United States and (2) the implementation of federal restrictions on tobacco products, including the Tobacco 21 law and the federal flavor ban of cartridge-based e-cigarettes. A deeper understanding of the key terms and emergent themes surrounding co-use may help inform communication campaigns surrounding harm reduction and access to treatment.

## Methods

### Data Collection and Sample

Tweets were collected using a Twitter firehose application programming interface through Brandwatch, a subscription-based social analytics software. Each tweet was selected for the dataset if (1) it was posted between October 2019 and September 2020, (2) it used at least 1 vaping-related term such as e-cig, vape, and e-liquid (see Table S1 in Multimedia Appendix 1 for full term list), (3) it originated from a handle (username) not identified as an organization or business, and (4) it originated from a geocode within the United States. This led to an initial sample of 63,008 tweets. The selected time period reflects a period of change in the United States, including both the emergence and initial peaks of COVID-19 and new federal restrictions on tobacco products (December 2019: Tobacco 21 and February 2020: federal flavor ban of cartridge-based e-cigarettes).

Similar to prior work, retweets (tweets originally composed by a different Twitter user and reshared by another user) and replies (responses to tweets that may or may not include the original tweet) were retained, as they were assumed to reflect an endorsement of the original post [50].

All tweets containing 1 or more alcohol terms were selected from the overall vaping tweet sample using the *grepl()* function in R (R Foundation for Statistical Computing) to form the final analytic sample of tweets mentioning both vaping and alcohol (N=6437 tweets). Alcohol-related terms used in prior topic modeling studies were reviewed by the study team, and a final set was selected for the current analysis based on a review of the content analysis literature, team consensus, and team review of initial test searches of the dataset. The final list of terms used was selected to ensure a broad search strategy reflected by tweets, which included use-related (tipsy and drunk), event-related (bar crawl and Thirsty Thursday), and product-related (beer and vodka) terms (see Table S1 in Multimedia Appendix 1 for full list) [46,47,51,52]. A subsample of tweets included in the final dataset was checked by the study team to confirm that they contained at least 1 vaping and 1 alcohol-related search term and that the content of the tweet was relevant to vaping or alcohol.

### Latent Dirichlet Allocation Modeling and Topic Description

We used latent Dirichlet allocation (LDA) to evaluate the major topics—or themes—among the sampled tweets. LDA is a machine learning topic modeling approach to identify naturally occurring topics. Model selection was based on the coherence score [53-55]. Specifically, coherence scores were evaluated for each topic count, and the highest score was selected to maximize model fit [56]. Tweets within each topic were then reviewed, and the labels for each topic were created by the research team, reflecting qualitatively emerging themes.

Tweet-level descriptive statistics were generated overall and by topic group. The top 15 most salient words overall are reported. The salience of a word is how informative it was for identifying a distinct topic within the model. More salient terms help differentiate across topics. For each topic, we report the top 15 most relevant terms (relevancy metric λ was set to 0.6 to assist topic interpretation as recommended by Sievert and Shirley [57]), sorted by frequency, along with mock tweets reflecting the topic theme. To protect user anonymity, no tweets were quoted verbatim. Instead, we created mock tweets reflecting the shared perspectives and common themes within each topic group.

### Metadata and Imputed Covariates

Each tweet contains metadata for the tweet itself and the Twitter handle (Twitter’s term for a username). These data are used as descriptive information and for demographic prediction.

We used deep learning techniques such as M3 demographic inference [58] and Bidirectional Encoder Representations From Transformers (BERT) [59] models to predict whether a Twitter handle was likely an individual or an organization, as well as the account user’s gender and age. Using machine learning approaches to predict demographics provides additional contextual data to tweets often missing from user accounts [45]. Given that vaping and drinking vary by age and gender, it is critical to understand potential variations in post activity and content by these key demographics [25,60,61]. A handle was required to have ≥50 tweets for gender and organizational prediction via M3 inference using information from the user’s Twitter bio, handle, username, and profile image (when available) [58]. M3 is a previously validated deep learning system to predict demographic information with an accuracy of 91.8% and 89.8% for gender and organizational status, respectively [58]. For the current dataset, gender was additionally validated against the gender prediction available in Brandwatch, and M3 inference was found to have a 93% agreement rate. Following prior applications of M3 inference, individuals with a probability score ≥0.5 for being a man were categorized as men, while those with scores <0.5 were categorized as women. Users with ≥0.5 probability score for being an organization were categorized as organizations, while those with scores <0.5 were categorized as individuals. Those categorized as organizations were not included in the analytic dataset, as previously noted in the *Data Collection and Sample* section.

For age prediction (<21 and ≥21 years), we built and trained a BERT [59] model using a dataset of 3000 prelabeled tweets and their last 100 tweets. The training model yielded high accuracy in age prediction (81%). We then used the trained age-BERT model to predict the age (<21 and ≥21 years) of the users using their last 100 tweets (see Multimedia Appendix 1 for additional details on model development).

Tweet sentiment was calculated using Valence Aware Dictionary for Sentiment Reasoning, a lexicon designed for quantitatively analyzing the sentiment of social media text, which has been previously validated and applied to Twitter data [62]. Valence Aware Dictionary for Sentiment Reasoning reviews text and automatically calculates a compound score ranging from –1 (most extreme negative) and +1 (most extreme positive). Scores were used to summarize average tweet sentiment overall and by topic group.

### Time Period Indicators and Analyses

Two federal regulations related to tobacco products were announced during the study period. The federal Tobacco 21 law (December 20, 2019) raised the minimum purchasing age in the United States from 18 to 21 years [48]. On January 2, 2020, a federal ban on flavored cartridge-based e-cigarettes was instituted (with exceptions for tobacco- and menthol-flavored products) [49]. We use the second regulatory change as the regulatory change indicator, given the close proximity of their implementation. Dates from January 2, 2020, and later were considered to have occurred in the post–regulatory change period, while dates prior to this date were coded as the pre–regulatory change period. Variation in the prevalence of each topic across regulatory time periods was tested via chi-square tests, and the resulting *P* value was reported.

We also examined variations in topic prevalence by whether they occurred before or after the declaration of the COVID-19 pandemic. Specifically, the World Health Organization declared COVID-19 a global pandemic on March 11, 2020. For this study, dates before versus on and after the pandemic declaration were considered pre– versus post–COVID-19 pandemic declaration periods, respectively. Variation in the prevalence of each topic by pandemic time periods was tested via chi-square tests, and the resulting *P* value was reported.

### Ethical Considerations

This study used publicly available Twitter data. Only study staff had access to the full tweets and associated metadata. All data were stored on password-protected devices. No tweets are quoted verbatim to ensure anonymity. There is no path from this manuscript or any supporting material to individual users, usernames, or tweets. The Boston University Institutional Review Board determined that studies using such data did not meet the definition of human participants and was thus exempt.

## Results

### Demographics

Roughly 10% of tweets that mentioned e-cigarettes or related terms included alcohol-related terms (6437/63,008, 10.2% after the exclusion of organizational accounts). Among the vaping-alcohol tweets where gender and age could be predicted, 61.9% (n=3448) of tweets were designated as likely originating from men, and 78.9% (n=3708) were likely posted by individuals ≥21 years of age (Table 1). The median follower count was 384 (IQR 141-1146), and the accounts followed were 505 (IQR 218-1170). Average tweet sentiment was largely neutral (80.1%). The majority of the tweets (n=5188, 80.6%) were posted in January 2020 or later, after the Tobacco 21 law and cartridge-based e-cigarette bans went into effect. This is slightly higher than would be expected if tweets were evenly distributed across months (75% of tweets would be from January 2020 or later). A little over half of tweets (n=3773, 58.6%) were posted in March 2020 or later, after the COVID-19 pandemic emerged in the United States. This is roughly what would be expected if tweets were evenly distributed across the time periods.

**Table 1:** Account characteristics among vaping-alcohol tweets overall and by topic.

Account characteristics		Overall (N=6437)	Topic 1: flavors and flavor ban (n=3334)	Topic 2: co-use discourse (n=1119)	Topic 3: availability and access regulation (n=1984)
**Gender^a^, n (%)**					
	Man	3448 (61.9)	1942 (65.9)	465 (48.7)	1041 (62.5)
	Woman	2119 (38.1)	1005 (34.1)	490 (51.3)	624 (37.5)
Age (>21 years), n (%)^b^		3703 (78.9)	1933 (75.8)	610 (82.4)	1160 (82.2)
Followers, median (IQR)		384.0 (141.0-1146.0)	396.0 (128.0-1351.0)	350.0 (168.0-658.0)	398.5 (150.0-1384.5)
Following, median (IQR)		505.0 (218.0-1170)	529.0 (202.0-1325.0)	375.0 (221.0-694.0)	592.0 (241.0-1459.0)
**Tweet sentiment^c^ (%)**					
	Negative	11.3	10	18.3	9.6
	Neutral	80.1	81.5	71.6	82.6
	Positive	8.6	8.5	10	7.8

^a^Among those where gender could be imputed, overall: n=5567, topic 1: n=2947, topic 2: n=955, and topic 3: n=1665.

^b^Among those where age could be imputed, overall: n=4700, topic 1: n=2549, topic 2: n=740, and topic 3: n=1411).

^c^Percentages based on average compound sentiment score across tweets.

### LDA Model Results

**Figure 1:**
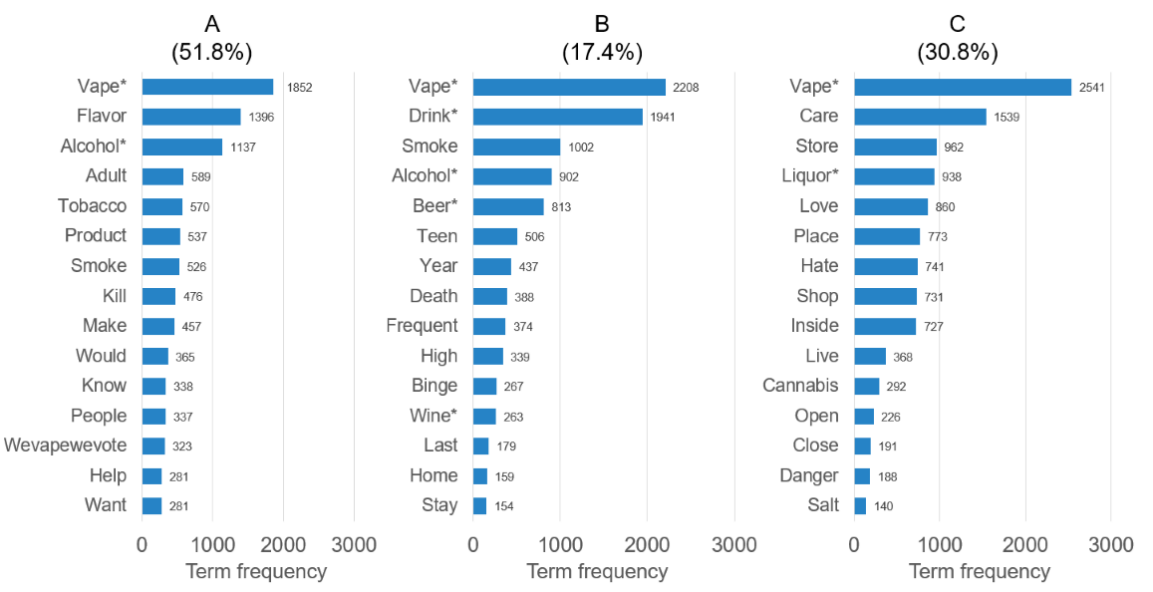
Top 15 most relevant terms ranked by term frequency among the 3 identified topics (N=6437 tweets): (A) topic 1: flavors and flavor ban (n=3334, 51.8%); (B) topic 2: co-use discourse (n=1119, 17.4%); and (C) topic 3: availability and access regulation (n=1984, 30.8%). * Indicates a term that was in the search term list for tweet selection (see Table S1 in Multimedia Appendix 1 for the full list).

A 3-topic model was the best fit for the data (coherence=0.39). Including additional groups resulted in less distinct topics, without improving model fit. Vape was the most common among the top 15 salient terms, followed by alcohol, drink, care, smoke, flavor, and store (Figure S1 in Multimedia Appendix 1). While COVID-19–specific terms (ie, pandemic and COVID-19) were mentioned, no specific COVID-19–related themes or terms were frequent or salient across topics. After qualitative examination of relevant terms and full tweets, the following topic themes were identified: (1) flavors and flavor ban (n=3334, 51.8% of tweets; Figure 1), (2) co-use discourse (n=1119, 17.4%), and (3) availability and access regulation (n=1984, 30.8%).

Within the flavors and flavor ban topic (topic 1), the most relevant and frequent terms were vape, flavor, alcohol, adult, tobacco, and product (Figure 1). Tweets tended to include arguments focused on how banning vaping flavors was incongruent with alcohol regulation where flavors are allowed and common. Tweets also often discussed the benefits of flavors for adults who made the switch from combustible tobacco products or espoused the opinion that e-cigarette use was less detrimental to youth’s health and safety than alcohol. This topic had the lowest proportion of tweets from individuals ≥21 years (76% vs 82% in other groups). Mock tweets representing common themes and tone within this group include:

We choose which flavor of liquor or candy to buy, why can’t adults have options when it comes to vaping flavors?

Banning vape flavors because kids use them makes no sense. Especially when alcohol is still available in flavors like bubble gum and vanilla.

Alcohol kills more youth a year than vaping ever has or will.

For the co-use topic (topic 2; n=1119, 17.4%), the most frequent words were vape, drink, smoke, alcohol, beer, and teen. Tweets often discussed plans or experiences related to using e-cigarettes and alcohol together in a positive light. This topic included tweets mentioning regulation, but these were less common compared with the other groups. Tweets within this group had a more even gender distribution (51.3% women vs approximately 35% in the other groups; Table 1). They were also more likely (compared to topic 1) to include individuals predicted to be older than 21 years of age (n=610, 82.4%). This topic also had the highest prevalence of both negative (18.3% vs roughly 10% in the other groups) and positive (10% vs roughly 8% in the other groups) sentiment, suggesting more polarization within this topic. Mock tweet examples include:

Listening to the Lumineers with my vape in one hand and a glass of wine in the other is my happy place

Just saw someone vaping in a non-smoking area, while throwing back a beer. What a flex!

I love vaping when I am drunk

The availability and access regulations topic (topic 3) represented roughly a third of tweets (n=1984, 30.8%). The most relevant and frequent terms were vape, care, store, liquor, love, and place. Tweets focused on regulation (similar to topic 1) but tended to include discussions of whether and how regulation restricts access. Some tweets supported regulations restricting youth access (ie, Tobacco 21) but expressed concern or anger that stricter regulations would limit access among legal-age adults. Tweets often mentioned age verification or limiting locations of sale as strategies rather than stricter regulations seen as moving toward “prohibition.” Alcohol regulations were held up as a comparison—either as an example of how complete bans of certain vape products may not work or to acknowledge the need to strengthen or enforce age verification processes and limit sale locations similar to alcohol. Statistics surrounding alcohol use and harm were also used for comparison, as were regulations and use prevalence of other substances, including cannabis.

Banning vapor products will not work, just like alcohol prohibition did not work.

Vapes should never have been available at gas stations. Allow products to be sold in age verified stores so adults have access while restricting access to kids—just like alcohol.

### Topic Prevalence Before and After Federal Tobacco Policy Changes

We next examined the distribution of topics by when in the regulatory change period they were posted. While the flavor ban topic (topic 1) represented over half of the tweets across the study period (n=3334, 51.8%, Table 2), flavor-related tweets were more common in the pre–regulatory change period prior to the changes to federal policy at the start of 2020 (Tobacco 21 and ban on flavored cartridge-based e-cigarettes) than in the post–regulatory change period (860/1249, 68.8% of pre–regulatory change period tweets compared to 2474/5188, 47.6% of post–regulatory change period tweets). Co-use discussions were more common in the post–regulatory change period (n=11, <1% of tweets prior to policy change vs n=1108, 21.4% of all tweets in the post–regulatory change period), while availability and access discussions (topic 3) were similar in prevalence across the 2 time periods (roughly 30%). Variations in topic prevalence across time were statistically significant across the 3 topics (*P*<.001).

**Table 2:** Variation in the prevalence of vaping-alcohol tweets during policy shifts and pandemic declaration overall and by topic.

Time period			Topic 1: flavors and flavor ban (n=3334), n (%)		Topic 2: co-use discourse (n=1119), n (%)		Topic 3: availability and access regulation (n=1984), n (%)		*P* value^a^	
Overall (N=6437)			3334 (51.8)		1119 (17.4)		1984 (30.8)		—^b^	
**Federal policy change status^c^**										<.001
	Pre–regulatory change period	860 (68.8)		11 (0.9)		378 (30.3)				
	Post–regulatory change period	2474 (47.6)		1108 (21.4)		1606 (31)				
COVID-19 pandemic declaration^d^										<.001
	Pre–COVID-19 pandemic declaration period	1628 (61.1)		273 (10.3)		763 (28.6)				
	Post–COVID-19 pandemic declaration period	1706 (45.2)		846 (22.4)		1221 (32.4)				

^a^Resulting from chi-square test for difference in proportions by time period across topic groups.

^b^Not applicable.

^c^Tweet was posted on January 1, 2020, or after.

^d^Tweet was posted on March 1, 2020, or after (World Health Organization pandemic declaration was on March 13, 2020).

### Topic Prevalence Before and After the Pandemic Declaration

Finally, variations in topic prevalence before and after the pandemic declaration were examined. Topic prevalence varied significantly in the pre– and post–COVID-19 pandemic declaration periods (*P*<.001). The availability and access topic (topic 3) was slightly more common in the post–COVID-19 pandemic period compared to the pre-period (n=1221, 32.4% vs n=763, 28.6%, respectively) as was the co-use topic (topic 2; n=846, 22.4% vs n=273, 10.3%, respectively).

## Discussion

### Principal Findings

In a sample of tweets mentioning e-cigarettes and alcohol, we identified 3 topics. Most tweets fell into 2 topics focused on vaping regulation while drawing comparisons to alcohol regulations. One additional topic including discussions of both alcohol and vaping (co-use) was also identified. Our findings demonstrated that while there were co-use discussions on Twitter, a large subset of comentions during this time focused on the regulation of these substances both before and after the implementation of federal policies related to tobacco. We also found that topic prevalence varied before and after key regulatory events as well as in the postpandemic period. Specifically, co-use discussions were most common after the pandemic declaration, which may suggest increased stress and substance use during a public health emergency. In addition, while flavor and flavor ban–related discussions were common across the study, they were more prevalent in the time period leading up to Tobacco 21 and the ban on flavored cartridge-based e-cigarettes. This may have reflected increased discussion prior to implementation as a way to express support or concerns surrounding the regulations.

### Comparison to Prior Work

These analyses add to our understanding of the social media discourse surrounding vaping regulation. Prior work examining Twitter discussion in response to the 2016 Deeming Rule by the Food and Drug Administration (FDA) on e-cigarettes found a rise in negative reactions after the announcement of the new regulations [35]. They also found that tweets often debated whether the rule would harm health or focused on how it would impact the new, emerging e-cigarette market [35]. Another more recent analysis that applied LDA topic modeling before and after the FDA’s flavor enforcement policy found similarly negative sentiment after implementation and discussions surrounding purchasing and accessing products after regulatory implementation [63]. Similar to these prior studies, tweets within the current analyses, which were collected during a period of changing policies, often debated the value of regulating e-cigarettes. In particular, how regulations implemented to protect youth would limit adult access to flavors and make purchasing products more challenging. Public health officials and policy makers should be aware of regulatory discourse in order to understand current public knowledge and sentiment as well as to contribute accurate and timely information to address concerns surrounding regulatory shifts. This may be particularly important in situations where posts are generated or elevated by the industry either directly or indirectly (ie, astroturfing) [64].

A novel finding was the use of alcohol as a regulatory comparator when reflecting on vaping regulation. While some tweets acknowledged alcohol regulation was beyond the FDA’s purview, the opinion that vaped nicotine products are more regulated than alcohol was consistently used to argue against vaping regulations. Additionally, use statistics among youth were commonly shared as evidentiary support. Specifically, some reported alcohol as a more common, harmful substance used by youth compared with e-cigarettes.

The prominence of access and regulatory commentary during this time period may have been in response to the new, evolving nature of regulations. One study analyzing tweets responding to an antivaping campaign found frequent objections to regulation as well as tweets touting e-cigarette health benefits [37]. In another study focused on marijuana use mentions—a substance experiencing regulatory change—calls for legalization were common [65]. Recent regulatory changes may have been more salient after the pandemic declaration due to challenges with access from business-related closures during this time period.

The identified co-use topic demonstrates an opportunity for those on these platforms (including youth audiences) to be exposed to content, which may normalize joint use. Common terms defining this subtopic included broad alcohol terms such as “alcohol” and “beer” but also terms that may be associated with heavy drinking such as “frequent” and “binge.” Our search strategy of including both general alcohol terms as well as common excessive drinking terms in the United States such as “blacked out” and “trashed” may have allowed us to more readily identify co-use tweets that would have not been captured with more standard drinking terms. These findings emphasize the importance of understanding cultural terms and specificity when collecting and analyzing social media data.

Counteradvertising efforts may be particularly useful during times of crisis and change as pathways for sharing accurate information in a timely fashion [44]. The unique time period of data collection may have also affected co-use discussions. Interestingly, the co-use topic was almost entirely restricted to the postpolicy period and was most common during the pandemic period. While regulatory shifts may have brought substance use discussions to the forefront, the early stages of the pandemic likely influenced co-use discussions. Over three-quarters of tweets within this topic were more specifically from the period after the COVID-19 pandemic was declared in March 2020. A prior study found alcohol use mentions related to coping during COVID-19 and addiction concerns during this time period [36]. With individuals spending time at home and living their social lives in virtual spaces, individuals during this time period may have increased their likelihood of sharing experiences related to substance use and co-use in particular. As LDA is a data-driven process, it is possible that if we had run the LDA separately across time periods for the regulatory change period or the COVID-19 declaration period, different themes may have emerged. Future research could adopt this approach to explore additional themes that may be missed within a larger dataset.

### Strengths and Limitations

This study has important limitations. First, analyses were limited to Twitter and to the text of tweets. Findings may not be generalizable to other social networks or more visual content (eg, videos and images), which are an important element of social media discourse. Second, although we included retweets, we did not explore key influencers that may drive content and were unable to examine the demographics of tweet viewers (ie, whether tweets reached underage youth). In addition, we did not distinguish between human and bot accounts. Furthermore, it is unclear whether users expressing frustration over regulations were industry funded or endorsed accounts. An analytic limitation is that LDA uses a “bag of words” method, which ignores word order and context. In addition, our focus on tweets mentioning both vape- and alcohol-related terms and the resulting smaller sample size likely limited the identification of additional topics. Finally, while we reviewed a subsample of tweets to ensure they included the relevant vape and alcohol search terms, we did not examine tweets for whether they were germane to vaping or alcohol use. It is therefore possible that although a term was used, the tweet was not focused on these topics.

This study also has several significant strengths. First, we used a data-driven approach to identify tweets mentioning both vaping and alcohol. We made no a priori assumptions about the number of topics or the thematic groupings and were able to quantify salient and frequent terms mentioned within identified themes. Additionally, we used state-of-the-art machine learning algorithms to predict account user demographics. Finally, we analyzed tweets from a time period, which included many changes in the United States, shedding light on how policy change and major national emergencies may shift social media discourse surrounding substance use.

### Conclusions

In conclusion, our study identified 3 distinctive topics across tweets including both vape- and alcohol-related terms. Specifically, a discussion of flavors and pending flavor bans was the largest topic subgroup, followed by a discussion of potential restrictions to e-cigarette availability and adult access resulting from regulatory changes, and finally, a portion of tweets included co-use of alcohol and vape products. While there were co-use mentions, tweets more often mentioned alcohol to make comparisons regarding how the substances were differentially regulated. Our findings demonstrate how the still-evolving landscape of electronic nicotine product policies influences social media discussion and debate. It also signals an opportunity to better understand and prepare for common concerns surrounding proposed regulatory changes—particularly as it relates to limiting youth access. A deeper understanding of social media discussions may inform health communication campaigns and policies focused on restricting underage youth’s access to substances and advancing youth’s health.

## Appendix

Multimedia Appendix 1 Additional methods details, alcohol and vaping terms list, and list of the top 15 most salient terms across the data set.

## Abbreviations

BERT: Bidirectional Encoder Representations From Transformers
FDA: Food and Drug Administration
LDA: latent Dirichlet allocation

## Data Availability

Analyses use publicly available posts from Twitter. The datasets generated and analyzed during this study are available from the corresponding author on reasonable request.
