# Supplementary material for: Concurrent Mentions of Vaping and Alcohol on Twitter: Latent Dirichlet Analysis
Source: J Med Internet Res. 2024 Nov 12;26:e51870. doi: 10.2196/51870 (PMC11599884; doi:10.2196/51870)

**BERT Vector for age prediction description:**

The BERT model is built on the Transformer’s encoder model that relies on multi-layer self-attention which allows the encoder to look at other words in the input sentence to compute contextual representations of each word in the sentence. To train the model, we inputted the user's tweets into the BERT model and then concatenated the BERT's last 4 layers of collective learning systems (CLS) to represent a tweet as a vector. Next, the average of all vectors of the tweets for each user was taken as the user’s vector representation. Vectors were then passed through a layer of fully connected neural network following by the Sigmoid activation function for classification. Code was adapted from the following github: https://github.com/WangKehanK/Twitter-User-Information-Prediction/blob/main/Twitter_User_Age.ipynb

**Table S1. Term lists for selection of vaping and alcohol-related tweets**

| **Substance** | **Terms** |
| --- | --- |
| Vaping | vaping, Vape, Vaper, Vapers, Vapin, Vaped, Evape, Vaporing, e-cig, ecig, e-pen, epen, e-juice, ejuice, e-liquid, eliquid, cloud chasing, cloudchasing, vapepen, vape pen |
| Alcohol | blackout, “blacked out”, “black out”, “blacking out”, “blacks out”, drunk, hammered, hangover, hungover, shitfaced, shit-faced, tipsy, drunk, “drink alcohol”, “drink booze”, “drinking alcohol”,  “drinking booze ”, intoxicated, sauced, buzzed, trashed, plastered, inebriated, shwasted, turnt, “Thirsty Thursday”, “Tequila Tuesday”, “Wine Wednesday”, “bar crawl",   beer, bar, alcohol, wine, vodka, liquor, tequila, whiskey, whisky, booze, cocktail, 6-pack, 12-pack, IPA, lager, stout, “hard seltzer”, pilsner, scotch, rum, brandy, cognac, liqueur, gin, vermouth, margarita, cosmopolitan, cosmo, “long island iced tea”, martini, “bloody mary”, “Moscow mule”, “whiskey sour”, mojito, daiquiri, “old fashioned”, mimosa, sangria |

**Figure S1. Top-15 most salient tweets across the sample ranked by term frequency**


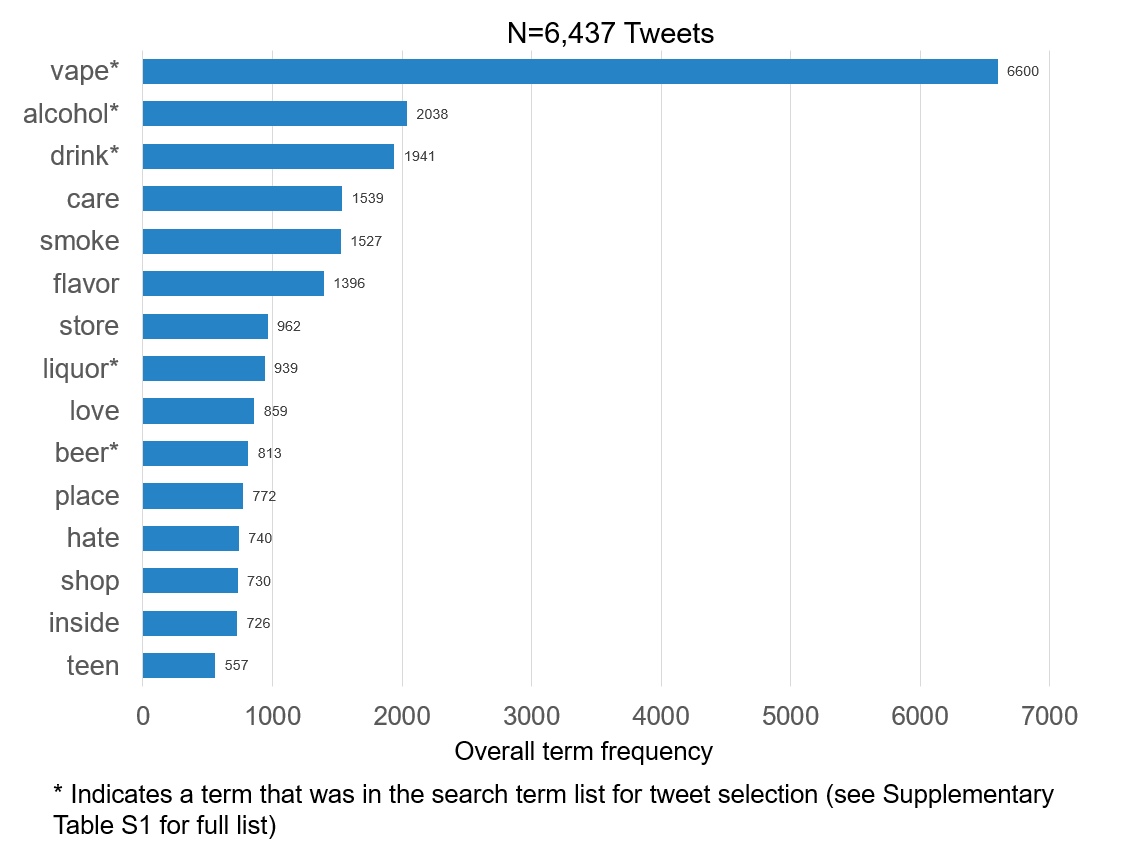

Supplement: Multimedia Appendix 1 [file jmir_v26i1e51870_app1.docx]
